# Endostatin shows a useful value for predicting failure to recover from acute kidney injury: some confounders to consider

**DOI:** 10.1186/s13054-020-2811-0

**Published:** 2020-03-17

**Authors:** Patrick M. Honore, Christina David, Aude Mugisha, Rachid Attou, Sebastien Redant, Andrea Gallerani, David De Bels

**Affiliations:** ICU Department, Centre Hospitalier Universitaire Brugmann-Brugmann University Hospital, Place Van Gehuchtenplein, 4, 1020 Brussels, Belgium

Jia and colleagues have concluded that plasma endostatin shows a useful value for predicting failure to recover from acute kidney injury (AKI) [1]. They studied two populations of patients with AKI following non-cardiac major surgery, with the primary endpoint of recovery or “non-recovery” from AKI. Patients classified as “non-recovery” from AKI in fact consisted of two groups, a cohort receiving renal replacement therapy (RRT) at day 7 and another cohort without RRT [1]. We would like to make some comments. Endostatin, the C-terminal fragment of collagen XVIII, is a cytokine with a molecular weight of 20 kDa [2]. It stands to reason that this small molecule can be easily removed by RRT as the cutoff point of filter membranes is about 35 kDa [3]. According to the authors, patients with renal recovery showed endostatin concentrations of 62.6 ng/ml, whereas patients failing to recover showed higher concentrations of 108.5 ng/ml [1]. Also, almost 20% of the AKI population received RRT for 7 days [1]. Considering that endostatin can be removed by RRT, the endostatin values in this group of patients may fall significantly [3]. This could give the clinician the false impression that the patient will recover from AKI. Accordingly, if endostatin is used as a predictive tool in the future, falsely low endostatin values in RRT patients could lead to a premature de-escalation of care for intensive care unit (ICU) patients. There has been no investigation of the performance of endostatin in patients who receive RRT. Therefore, we believe there is a critical need for a future study with a focus on the performance of the currently known sepsis biomarkers in patients who receive RRT [4]. As noted by experts in endostatin, there is not enough evidence to date to support the use of endostatin measurements in clinical practice [5]. RRT is a good example of one of those conditions [5].

## Authors’ response

Endostatin is a useful biomarker to predict recovering failure from acute kidney injury

Hui-Miao Jia, Wen-Xiong Li

We appreciate the comments of Honore et al. on our article.

In this study, endostatin plasma concentration was detected immediately after acute kidney injury (AKI) diagnosis and before the renal replacement therapy (RRT) started and showed a useful value to predict the recovering from AKI failure. Early prediction aims to enable individual treatments and effective interventions that may improve clinical outcomes. Endostatin is a 28-kDa molecule that can be removed by a high flux membrane [6]. Different filtration membranes with different bore diameters can remove different sizes of a molecule. The clearance of the biomarker is significantly dependent on the molecule size and its sieving coefficient. This article did not evaluate the performance of the biomarker in RRT; thus, we did not analyze renal recovery according to plasma endostatin concentration during RRT. Further studies may be conducted to explore this issue in the future.

## Data Availability

Not applicable.

## Abbreviations

AKI: Acute kidney injury
RRT: Renal replacement therapy
ICU: Intensive care unit
